# Identification of Safety-Related Opinion Leaders among Construction Workers: Evidence from Scaffolders of Metro Construction in Wuhan, China

**DOI:** 10.3390/ijerph15102176

**Published:** 2018-10-04

**Authors:** Chaohua Xiong, Kongzheng Liang, HanBin Luo, Ivan W. H. Fung

**Affiliations:** 1 Department of Construction Management, School of Civil Engineering and Mechanics, Huazhong University of Science and Technology, Wuhan 430074, Hubei, China; xchxch@hust.edu.cn (C.X.); luohbcem@hust.edu.cn (H.B.L.); 2 Department of Civil and Architectural Engineering, City University of Hong Kong, Hong Kong, China; ivan.fung@cityu.edu.hk

**Keywords:** construction safety, scaffolder team, social network analysis, opinion leader, unsafe behavior

## Abstract

This study aimed to reveal opinion leaders who could impact their coworkers’ safety-related performance in Chinese construction teams. Questionnaires were distributed to 586 scaffolders in Wuhan to understand their opinions about influencing their coworkers, serving as the foundation for a social network analysis to identify the potential opinion leaders among workers. A further controlled trial with the identified workers was conducted to select real opinion leaders by comparing their influence on others’ safety-related behavior, followed by an association analysis to profile these opinion leaders. Two main sources of opinion leaders were identified: foremen and seasoned workers. Implementing interventions through opinion leaders resulted in better safety-related behavior performance. Furthermore, compared with education level, the association analysis results indicated that one’s practical skills and familiarity with respondents was more important in the formulation of opinion leaders. This research introduces the concept of opinion leaders into construction safety and proposes an approach to identify and validate opinion leaders within a crew, thus providing a tool to improve behavior promotion on sites, as well as a new perspective for viewing interactions among workers.

## 1. Introduction

The construction industry is widely known as one of the most dangerous industries. Research from many nations has shown that construction workers still suffer from a higher proportion of occupational injuries and fatalities compared with other industries [1,2,3,4]. Therefore, numerous studies have been conducted on various topics from the individual level, such as worker behavior and competency [5,6,7], to enterprise-level work, such as focusing on the safety costs of construction firms [8,9]. As a trend in the organizational level studies, interrelationships among workers and different roles in construction safety on sites have been considered [10]. Harmonious working relationships among the foreman and co-workers were found to result in positive safety performance for the entire team [11,12]. Researchers also focused on the special roles among coworkers on sites. Fang et al. implied that the foremen onsite are an important factor influencing workplace safety management [13]. Other researchers share this opinion, as they argued that the foreman plays a critical role in accident prevention [14], whereas some studies examined the impact of foreman behavior and perception in terms of daily workplace safety management [15,16].

However, most previously published articles focused on the foremen on site, which is partly attributed to the fact that the foreman is the most obviously influential role in a working team, whereas few studies have been published on other potentially key roles in a crew. There is strong evidence showing that co-workers affect each other. A meta-analysis based on 161 independent samples and 77,954 employees indicated that an employee’s perceptions, attitudes, and performance are influenced by their workmates even after accounting for managerial impacts [17]. A similar conclusion was reached in a study involving 991 blue-collared workers, confirming the existence of a mediating role among co-workers that more strongly influences safety behaviors compared to that of top management and supervisors [18]. Considering the nature of mobile workers, the temporary organization on-site, and complex subcontracting in the construction industry, construction workers are prone to disconnect with top management and are more connected with their crews [19]. Therefore, it is worthwhile to attempt to reveal whether potential special roles exist among the construction crew, and if the individuals in these roles are able to significantly impact their crew’s behavior and safety performance during operation.

To reveal whether potential special roles exist in an organization, social network analysis (SNA) has been proven to serve as an effective approach in prior articles. The fundamental assumption of this analysis is that the level of communication, which refers to the frequency of information and knowledge exchange in a group, affects the performance of the whole project and each participant [20]. Additionally, Zohar’s study supported this assumption. The findings demonstrated a positive relationship between the increase in safety-related communication intensity and safety-promoting behaviors [21]. A recent study applied SNA to analyze the impact of communication on the construction safety climate in China, which indicated that those who are responsible for safety management (e.g., safety supervisor or site construction manager) represent a higher density in the communication network [22]. As a result, it can be inferred that those who are in potentially special roles in a crew are more likely to show a higher intensity in terms of safety communication, thus providing more opportunities to influence coworkers through exchanging knowledge and information [23]. Furthermore, to better understand these influential members, we introduce the concept of the opinion leader to describe their influence in a crew. In the social network analysis field, the opinion leaders are defined as people who are ‘central’ in their social group at work [24] and have the capacity to influence the opinions, thoughts, and behaviors of others in their community [25]. This concept was chosen not only because it satisfies the hypothetical effect of these special members in a community but also due to the importance of opinion leaders in behavior change promotion [25], which provides a tool to validate the hypothesis. In other words, with regard to safety performance, it is reasonable to deduce the existence of key roles in a crew if the performance of the intervention by selected members precedes that of others within the group.

This study aimed to investigate the potential special roles in construction teams in China through social network analysis and examine their influence on the safety performance of their groups from the perspective of behavior change promotion using opinion leaders. A method is proposed for identifying influential members who have a more significant influence on coworkers in terms of safety performance, and an association analysis is then conducted to gain deeper insight into the characteristics of the selected members.

## 2. Literature Review

The SNA was initially proposed as an analytical method by Moreno and Jennings, with the aim to delineate the communication relationships among members belonging to one community with graphics and metrics [26]. Following the initial data collection, statistical analysis and sociograms were incorporated into Moreno’s approach to provide a more comprehensive interpretation, thus building a prototype of subsequent SNA-related analysis, using SNA diagrams and related statistics [27]. In practice, SNA is used for identifying people who are most central within a community [28], as well as for revealing the underlying mechanisms and dynamics driving the exchange of information and knowledge [23]. A systematic review based on 191 articles showed that social network analysis-related techniques are the most popular methods for identifying opinion leaders [25]. In comparison with other methods (e.g., self-based method, positional approach, and judge’s rating-based method), social-network analysis-related methods have been shown to be more objective with less selection bias. With this method, the entire communication structure involving all members of the community is mapped, and thus various centrality techniques and optimal matching strategies can be used to identify opinion leaders [28,29]. At first, the fields of healthcare and public health were the most active in their use of SNA to identify opinion leaders. Studies on primary care used SNA to outline the network of influential discussions among physicians to identify their roles and relationships with others within the network [30]. Similarly, SNA was also introduced to understand the mechanism for knowledge sharing among medical staff [31]. The concept of opinion leaders in the social network has similarly been extended to other fields such as education, telecommunications, and marketing [32,33,34].

The common idea used by relevant research involves constructing a network using SNA in different contexts of communication, which is then used in sharing knowledge and perception. Then, the opinion leader is identified based on centrality derived from the different agreed-upon threshold of nominations received from other members in the network. This leader is expected to act in a more important role in his/her community with respect to the influence on others’ attitudes, perceptions, and behaviors. Many authors confirmed this hypothesis and extended this perspective to behavior intervention through opinion leaders. It has been proven that physicians are prone to ask colleagues with greater expertise and experience for advice about the care of their patients [30]. Sales et al. argued that physician opinion leaders can extend their influence by filling holes in their network or strengthening information ties, thus promoting dissemination and translation of knowledge [31]. Starkey developed a SNA-related approach to identify influential young people to deliver smoking prevention among their peers. As a result, the intervention through opinion leaders resulted in a more effective diffusion of health promotion messages among adolescents [33]. The influencing effects of key users on others’ opinions are also used in marketing. The intervention of key users in a social network can simulate the spread of opinions, as well as influence other members’ new product adoption decisions [34]. With the development of online communities and database techniques, some authors attempted to identify the opinion leaders among large samples and the resulting complex networks. A Russian study determined what drives the formation of latent discussion communities by identifying opinion leaders and shared topics from a co-commenting network including 520,000 comments and 4.5 million edges [32].

With regard to safety research at the crew level in construction, SNA has primarily been implemented to serve as a tool to map communication patterns in projects and obtain resulting indicators, which are regarded as being related to knowledge sharing, safety performance, and efficiency [22,35,36,37]. A study on small crews (5–12 members) showed that social density in a network can be used for distinguishing the high- from the low-performing teams, as safety performance of a crew is partially related to the frequency of safety communication within teams, as well as the number of workers who are able to act as a link for sharing safety knowledge [38]. Their subsequent research provided support for this hypothesis through an exploration of the characteristics of multilingual safety networks, which showed that crews with relatively weak safety performance tended to have clear and disparate subnetworks distinguished by language and high turnover rates. Among these multilingual teams, bilingual workers often formed the core of a network that connected disparate groups of individuals [36]. Allison and Kaminsky further extended the previous work by providing demographic features to consider when mapping a communication network and identifying the influential roles within the crew. Their findings demonstrated the impact of sex on crew safety communication in terms of central roles in network and communication patterns, which produced different safety performance in comparison with homogenous all-male crews [35]. Similar research was also conducted in the context of the Chinese construction industry. Liao et al. characterized team communication in China using SNA and then discussed the association between different communication structures and workers’ safety awareness. In this study, safety supervisors and site construction managers were shown to serve as driving forces that could focus workers’ attention on safety management and improve the safety climate [22].

However, in addition to the findings derived from SNA methods, construction worker behavior has been studied from different perspectives. Some researchers extended social norm and behavior in worker’s absence behavior, indicating that absence behavior is not only determined by an individual’s disposition or personal situation but also controlled by related beliefs and values shared in their group [39,40]. Ahn and Lee subsequently conducted an in-depth study using social identity theory to explain the changes in personal standards regarding safety behavior in the United States [41]. A similar study was carried out in Denmark [42], which demonstrated that interactions within group members lead to a change in social norms and the resulting behaviors of workers. Some other studies focused on the potential key people involved in this kind of interaction. Albert and Hallowell investigated hazard recognition and communication level data from 18 active construction crews in the United States and provided evidence for the importance of the role of supervisors for improved safety performance [43]. Fang et al. also found similar results from an empirical study of the Hong Kong construction industry, indicating that supervisors impact group-level safety climate, which in turn affects workers’ safety behaviors [44]. Another study reconfirmed the existence of key people who affect safety, and even argued that the interactions between workers should be an important consideration when planning safety measures on projects [45]. Despite the importance of interdependence and communication in construction crews being emphasized in several studies, research analyzing social interactions, network properties, and their relationship to safety-related behavior is still inadequate [43]. Furthermore, most current studies about the key people involved in interactions have concentrated on the foreman and supervisors, whereas research that focuses on the potential influential role among general workers is lacking.

As indicated by previous research, SNA provides an approach for converting complex social communication into a visual format, including quantitative indicators that can be used to evaluate interpersonal relationships. The resulting network efficiently reflects the paths of knowledge transfer and information exchange among members, especially for colleagues or co-workers who work in similar environments. Information agents defined by SNA metrics emerge, and these individuals are expected to act in a more influential role in terms of the opinions, attitudes, and motivations of others, resulting in changes in determination and behavior during operation. Considering the complex nature of the construction industry, the formation of influential roles in a construction crew depends not only on the locations in the managerial hierarchy but also on various other factors [19]. Therefore, the existence of such nodes derived from SNA provides the foundation for the first research question of this study: In a construction crew, are there potentially special roles other than foremen in first-line workers on sites that are located in a central position in the safety-related communication networks?

However, even if such roles exist among first-line workers, these information agents may not necessarily have a more significant influence on their coworkers’ perception and behavior. Therefore, the concept of opinion leaders was applied to examine the effects of these information agents, as well as to provide practical implications to the expected findings. Previous research in various fields suggested that intervention using opinion leaders helps to change norms, via the adoption of new perceptions, and promotes behavior modification, although there is an obstacle to overcome in terms of validating the effectiveness of the intervention. With the help of a video surveillance system on sites, a qualified pro-active behavior-based safety indicator [46] was implemented to quantitatively evaluate the change trend of safety-related behavior at the crew level. As a result, the introduction of the concept of opinion leaders and corresponding validation methods offered an opportunity to answer another research problem: Do the identified information agents have a more significant influence on their co-workers’ safety-related behavior in daily operation?

This paper hypothesizes that some seemingly ordinary workers may occupy central locations in terms of safety-related communication during construction operation. The authors further expected that a more targeted intervention on these workers would lead to the promotion of behavior changes of the whole team. Inspired by previous research, we conducted one of the first attempts in the context of the Chinese building sector to extend the SNA method and the concept of opinion leaders to the front-line worker, aiming to identify and characterize key people who are influential in safety-related information communication, and to validate their importance in promoting efficient and frequent interactions among workers through an intervention program targeted toward these members.

## 3. Research Methods

### 3.1. Setting and Participants

Data were collected from scaffolder crews working on 20 metro construction sites in Wuhan, China. The participants were assigned to a control group or intervention group. In the intervention and control groups, behavioral interventions on unsafe behavior were conducted on an equal proportion of workers. The intervention group only intervened with opinion leaders, whereas the control group intervened randomly with workers.

As a result of increasing traffic congestion and population and the need to improve transportation infrastructure, Wuhan, Hubei province, China, is completing large-scale metro construction. Managers want to reduce workers’ observable unsafe behaviors. Therefore, a more effective reaction system for behavioral risk was established to meet the goals of safety management. Meanwhile, many safety accidents occur due to unsafe behavior of scaffolders, resulting in a high scaffolder injury rate during the metro construction process. Therefore, we chose the scaffolders as the research subjects in the metro station construction process. These samples were mainly sourced from 20 stations on two lines (Lines 3 and 6), provided by the construction firms after obtaining the consent of the metro company and the construction enterprise.

The sample had 586 scaffolders (93% men, 7% women). From Table 1, most of the participants were junior high school educated (75%), were on-site workers (95% workers, 5% foremen) had worked 20 years or more (69%), and had worked at the same team for less than three months (35%).

### 3.2. Sociometric Assessment to Determine Each Network’s Social Leader

A questionnaire developed based on previous research was distributed to workers [35,36,47]. The questionnaire consisted of two parts: the first part included several general demographic questions including sex, education level, status in the crew, working experience, and team tenure, whereas the other part contained three questions: (1) Have you received advice on occupational safety from this person? (2) With respect to safety during operation, have you been influenced by this person’s words or behaviors? (3) If you have questions concerning safety operation in the future, will you ask for this person's advice voluntarily?

A list of their crew members was provided to each respondent, and they were asked to check a box beside each name that corresponded to each of the three abovementioned questions. Except for the first two general questions in the opinion leader survey, the “future advice” item was also added to measure behavioral intentions, which had been shown to complement measures of past behavior in predicting future conduct [47]. As a result, a table that reflected the number of times that a crew member was checked off by their coworkers was obtained. We essentially created three sets of nomination scores for each scaffolder. Afterward, considering the different scale of crews, for each participant within the same crew, their nomination frequency for the three questions was normalized by dividing the number of nominations received by the total possible number of nominations. The second part of the questionnaire provided a foundation for the following SNA to identify the possible opinion leader. For instance, in a crew of 21 workers, if a member was selected by four coworkers as someone who has delivered safety-related advice to them in the past, then their nomination score for the first item would be 0.2 (i.e., 4/(21 − 1) = 0.2).

### 3.3. Social Network Intervention Procedures

After the completion of the questionnaire survey, we used the social network method to identify potential opinion leaders in terms of unsafe behaviors, but we were still uncertain if we would be able to improve construction site safety through such opinion leaders. Therefore, a controlled trial was designed to conduct interventions in an intervention group and a control group. Behavior-based safety (BBS) is an approach that can be used to modify the behavior of people to undertake work more safely [48], which includes the following steps: (1) unsafe behavior is listed, (2) workers’ unsafe behavior is observed and its frequency is recorded, and (3) feedback is given and their unsafe behavior is modified. Therefore, the authors first confirmed the list of unsafe behaviors in the construction process. Several safety standards and operating instructions in China were used as references to list partially unsafe behavior: Standard for Construction Safety Assessment of Metro Engineering (GB 50715-2011) and Quality and Safety Check Points of Urban Rail Transit Engineering (2011). From the data sources presented above, a total of six unsafe behaviors about scaffolders were identified and classified including: (1) scaffolder worked in sleety weather without wearing non-slipping shoes, (2) scaffolder worked more than 2 m high without wearing a harness, (3) scaffolder climbed up and down the scaffold without protection, (4) scaffolder stood in a location without sufficient scaffold floor, (5) scaffolder threw member bars from height in the process of dismantling, and (6) scaffolder stacked too much material on the scaffolds. After determining the unsafe behavior list, we used video surveillance to observe behaviors of the scaffolders. The manner in which we collected data on worker behaviors is explained in detail in the next section. Finally, safety information was provided to workers; for example, workers were subjected to daily safety inspections and photos that reflected the unsafe behavior on site were used regularly to encourage the workers via intelligent mobile In addition, the intervention group only intervened using opinion leaders, whereas random workers in the control group received interventions. The whole process of intervention lasted four weeks. During this period, workers were encouraged to communicate safety information to prevent unsafe behaviors.

### 3.4. Worker Behavior Data Collection

In order to evaluate the effect of network intervention, we observed unsafe worker behaviors and recorded their frequency. The research used video surveillance to collect unsafe worker behavior and this method proved to be effective [49]_ENREF_21. To ensure the sampling video would be representative of the whole working activity, the study adopted a random sampling method to collect video. In addition, this study performed several tests to determine how to sample the video clips that were to be analyzed [50]. The following parameters were considered: duration of a single observation, sampling frequency, and number of cameras visualized on the screen at the same time.

For facilitating the observation and recording of behavior data, a specific checklist to support the analysis of video records and data collection was developed (Table 2). For each of the issues critical for safety, the checklist provided a list of unsafe behaviors on a metro construction site.

The data collected through the checklist were also intended to be used to quantify the following safety indicator: the proportions of safe behaviors and unsafe behaviors (total or detailed by category) registered during a period of time.

## 4. Results

### 4.1. Identification of Potential Opinion Leaders

**Figure 1:**
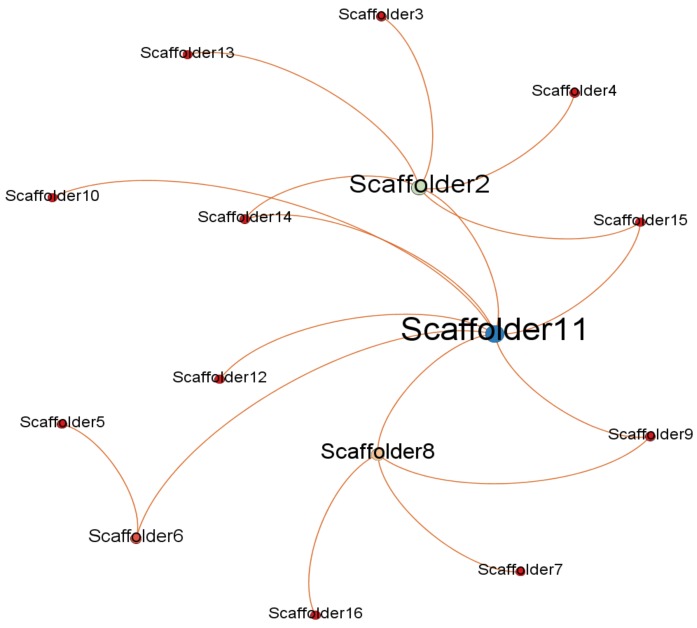
Project 1 social network structure.

**Figure 2:**
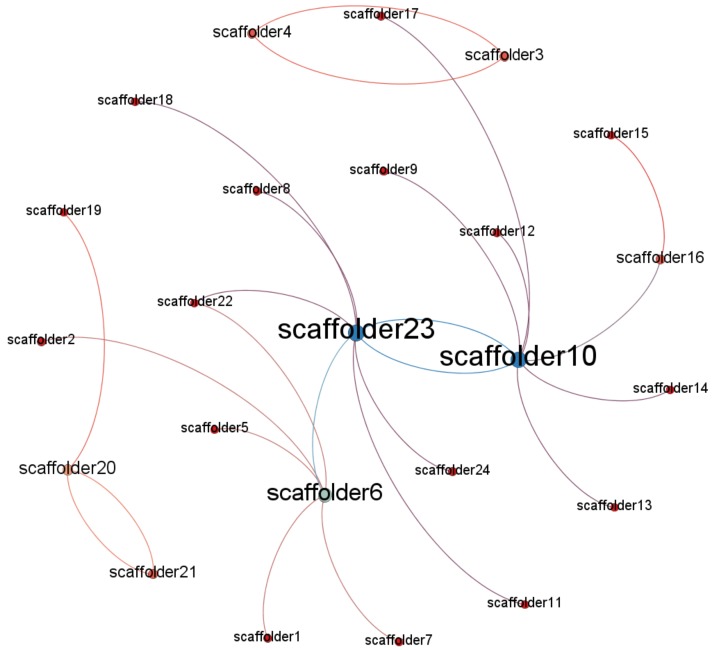
Project 2 social network structure.

**Figure 3:**
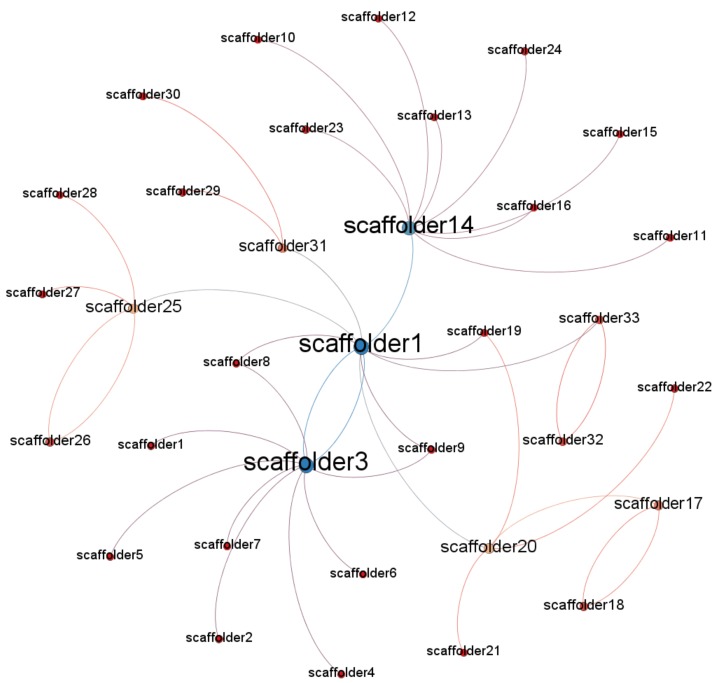
Project 3 social network structure.

To identify potential opinion leaders with the social network method, the most important and frequently-used concept is network centrality. Network centrality measures the structural importance of actors and indicates which actor is in the center of the network. In order to use the social network method to identify opinion leaders related to unsafe behaviors, Gephi was used to map the networks consisting of nodes and edges. Gephi is a visualization tool that automatically generates and reveals patterns in the structure of each communication network [51]. Considering the directivity of the data collected by the questionnaire, the graphics type selected was “directed”. Figure 1, Figure 2 and Figure 3 show that there are existing networks of unsafe behavior among scaffolders, and the size of the node reflects the different influences among workers.

By observing the network of Project 1, depicted in Figure 1, the network is centralized around the foreman, who has the highest degree value. However, there are also some nodes with higher degree values than others. Figure 2 shows the social network structure for Project 2. The number of players involved in construction is higher than in Project 1, which is mainly related to the type of the project. Similar to Project 1, there are some nodes with higher degrees, including the foremen and some workers. However, some nodes, like scaffolder 3 and scaffolder 4, are not easily reachable within the network, which displayed a high level of segregation. Finally, Figure 3 shows the social network structure for Project 3, which has more nodes than Project 2. In addition, the social network structure for Project 3 is quite similar to the network structure of Project 2, which also had some nodes that were not easily reachable within the network.

Social network analysis was carried out to evaluate the performance of each network against specific measures. Results were computed using Gephi according to the different sizes of the networks, which are summarized in Table 3. The mean composite nomination scores were 1.02, 1.04, and 1.06 for the different network scales of Projects 1, 2, and 3, respectively (Table 3).

To determine which worker in the 20 subway stations construction project was an opinion leader, we selected an arbitrary composite nomination cut-off score: 0.20. This score was equivalent to receiving over 20% of the nominations of participating peers, which was taken to mean substantial peer respect/support. Using the 0.20 cut-off, all subway station projects had at least one identifiable potential opinion leader in addition to the foreman (Table 4), providing an answer to the first research question. Meanwhile, we found that when the social networks scale is large, the percentage of opinion leaders was lower.

### 4.2. Effects of Intervention on the Behavioral Outcome

As shown in Table 5, several aspects were analyzed for the control group and the intervention group. The results show that no differences (*p* > 0.05) were found between the intervention and control groups, indicating that the randomization was successful.

In this section, the authors introduce the results of a random reference test. Before discussing the results of the behavioral studies, we considered the potential bias of observers. In the present research, the main recommendations available in the literature for reducing this risk were adopted [50,52,53]. A precise protocol for the classification of observational items was adopted with the aim of increasing the objectivity of the process and the robustness of the research results. Inter-rater reliability was assessed at the beginning of the project with a score of 96, which is well above the threshold recommended in the literature [52]. Finally, the focus of our analysis was not on absolute numbers, but instead on assessing trends and changes over time.

In order to observe further quantification of the scaffolder behavior result, each unsafe behavior record was converted to a percentage. The Safety Index (SI) [46] was adopted to test the unsafe behavior using the following formula:

(1) $$\;SI=\frac{N_{2}}{N_{1}+N_{2}}\;$$

where *N_2_* is the number of observed instances of safe behavior, *N_1_* is the number of observed instances of unsafe behavior, and *N_1_ + N_2_* is the sum of all instances of the previously specified safety-related behavior.

**Figure 4:**
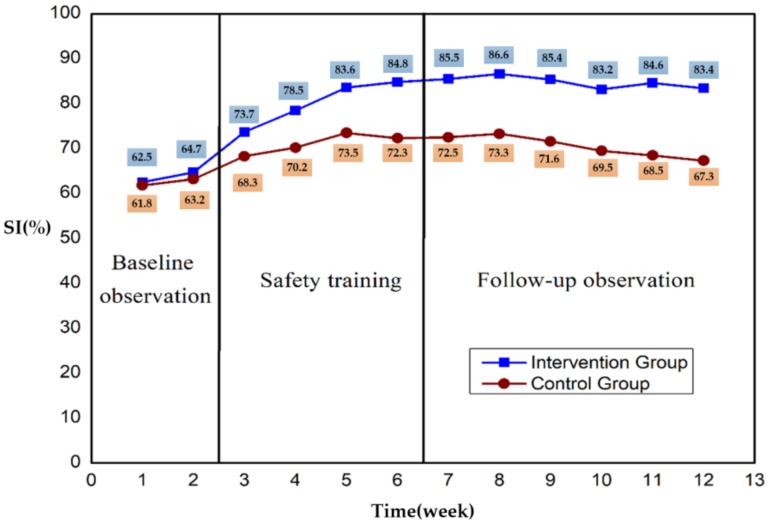
Safety Index (SI) change trend chart for the control group and intervention group.

Four weeks of safety training and six weeks of behavior observation were carried out for the control group and the intervention group. As shown in Figure 4, the total SI scores of the intervention group and the control group during the two-week baseline were 62.5 and 61.8, respectively. After safety training, SI increased to a maximum of 86.6 and 73.3, respectively. Without further intervention, this SI decreased during the follow-up observation phase to 83.4 and 67.3 at the end of the 13th week, respectively. Finally, the SI improved 20.9 and 5.5, respectively.

**Figure 5:**
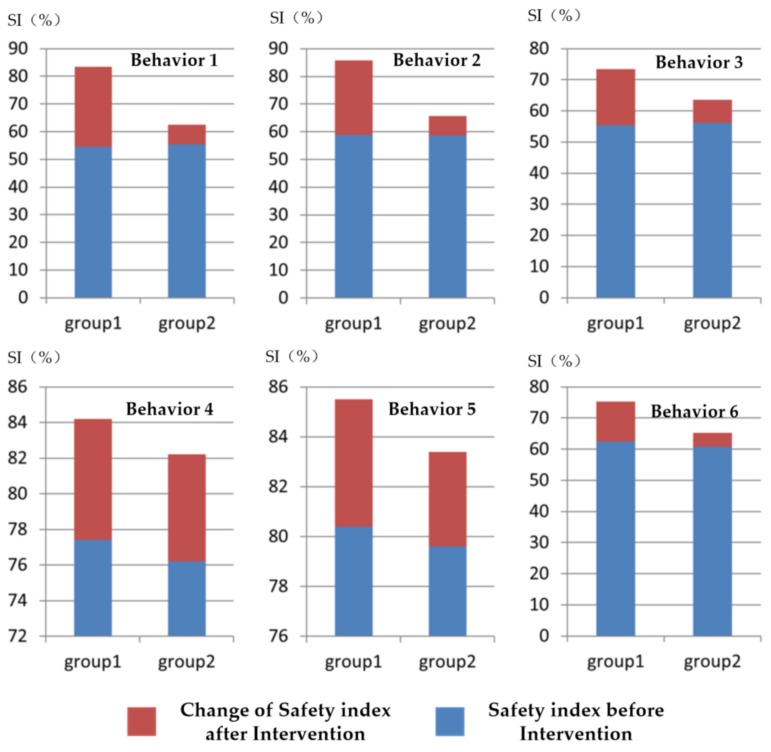
Safety Index (SI) change in six unsafe behaviors for two groups after intervention. Group 1: intervention group; Group 2: control group. Behavior 1: working in sleety weather without non-slipping shoes; Behavior 2: working more than 2 m high without safety harness; Behavior 3: climbing up and down the scaffold without protection; Behavior 4: standing in a location without sufficient scaffold floor; Behavior 5: throwing member bars from height during dismantling; and Behavior 6: stacking materials on scaffolds.

In order to assess possible differences in behaviors among six kinds of unsafety behavior, one additional figure was drawn by changing the aggregation level of all the dataset (Figure 5).

To further analyze the improvement in all kinds of unsafe behaviors, the statistical significance of the group data was analyzed. The analysis results in Table 6 show that the intervention group had a significantly higher improvement in four of the six unsafe behaviors, thus partially addressing the second research question.

### 4.3. Factors Associated with Opinion Leadership Status

In order to help managers to identify potential opinion leaders more quickly, the research attempted to find potential characteristics of opinion leaders through association rules. Using association, we identified the interdependences between the factors and the influence of workers. The factors included (a) sex, (b) education level, (c) status, (d) years of work experience, and (e) team tenure.

A Boolean matrix was constructed by using the attribute information of the workers, as shown in Table 7. Number 1 in the matrix indicates the occurrence of the attribute, and 0 indicates that the attribute did not appear.

In our study, the rules were filtered by support and confidence, where support is the percentage of the entire data set covered by the rule, and confidence is the proportion of the number of examples that fit the right side among those that fit the left side. To identify strong associations, threshold values for support (S) and confidence (C) were set as follows: S ≥ 5, C ≥ 60. The characteristics of the workers were set as the leading items, and the worker influence was set as the successor.

As shown in Table 8, we found that the work experience of opinion leaders was greater than others. The foremen in each group were more likely to be opinion leaders, and women, who were fewer in number, did not show the potential to be opinion leaders. In addition, in comparing team tenure lengths, the results showed that the difference between opinion leaders and others was obvious.

## 5. Discussion

This study’s findings indicate that a more focused intervention toward the selected opinion leaders within crews can produce superior results in terms of worker safety behavior on sites. In the context of our study, the opinion leaders in the construction teams exerted their influence by providing advice or communicating with workmates during operation, and they were therefore identified as information agents by SNA, nominated by their workmates as sources of safety-related advice, and provided a benchmark of daily operation. However, the number of workers who could fulfill the threshold value (0.20) decreased as the number of crew members increased, resulting in more complex social networks being obtained, even if the proportion of information agents remained consistent. This implies that the influence of opinion leaders is limited; the scale of workers under one’s influence will not change as much as the social network to which they belong expands. This may be attributed to the high intragroup closure among construction crew members and the lower level of intergroup care about coworkers’ safety, especially for a large crew size [54,55]. The data in Table 3 show another difference in the decline with respect to the proportion of opinion leaders between the nominated foremen and workers when the scale of the network increases. In other words, for a complex social network in our study, the workers were more likely to ask the designated person for advice instead of discovering someone from amongst workmates. Previous research in a manufacturing facility provides an explanation by linking safety-related behavior with the perceived organizational support and leader-member exchanges [56]. In addition, considering the temporary organization and short tenure for workers on sites, it may be more difficult to form a stable safety-related communication network for construction workers in a large project.

The second finding provides suggestive evidence that the influence of opinion leaders on some certain behaviors was not as notable as expected. Compared with other mentioned unsafe behaviors in Table 2, the interventions against two kinds of behaviors (standing in a location without sufficient scaffold floor and throwing bars to coworkers from height in the process of dismantling) had no significant differences between intervention group and control group. One might presume that a ceiling effect may prevent further optimization of worker behavior, allowing for relatively higher initial SI values (SI > 75%) for these two behaviors. Similar effects also can be seen in Figure 2, where the SI index of the control group more obviously increased in the first two weeks after the intervention was introduced. This finding could have been due to the better safety performance of selected potential opinion leaders, thus complicating the ability to create an instant result when the intervention was introduced to those with a relatively high initial SI index compared with that of the randomly selected participants in the control group. However, as the continuous intervention proceeded, the intervention group outperformed the control group, implying that the wider influential scope of key roles in crews resulted in behavior change promotion at the team level.

This research also revealed two typical opinion leaders: foremen and seasoned workers. Despite the relatively stable proportion of nominated foremen when the scale of the network increased, the foreman was also the only type of opinion leader that did not need to meet the tenure requirement (i.e., six months) according to our research. This may be because the foreman is generally accepted as a central role in a crew and is authorized to take compulsory measures (e.g., rewards, supervision, and penalty) to control the crew [57], which means a wide and immediate effect within crews compared with nominated workers. With respect to the nominated workers, we did not expect that the experienced worker would be another source of opinion leader. As an extensive and in-depth study based on previous findings [43,44]., except for the foremen, this study revealed the influence of seasoned front-line workers in safety-related information transfer and communication, as well as in other worker safety behavior, thus contributing to the body of knowledge about social interaction among workers and resulting changes in worker behavior. This study also provides one possible explanation for the insignificant association between supervisor or foreman centrality in networks and hazard recognition in previous research [43]: construction workers typically learn how to perform tasks from the seasoned workers [58], so the journeypersons instead of foremen or supervisors provided the most guidance about safety to other workers in the crew, especially to the apprentices [59]. In this regard, this kind of opinion leader may exert their influence by providing safety-related guidance and interpretation. However, all the opinion leaders selected from the experienced workers worked at one site for at least six months, which may be a precondition for the formation of this kind of leader. Surprisingly, a strong association pattern was not observed between education level and the nomination of opinion leaders in our results. Education level was emphasized as an important influencing factor in individual safety behavior [60,61], but it seems that one’s practical skills and familiarity with the respondents played a more important role for the formulation of opinion leaders at the current stage. Nevertheless, most current older but experienced migrant workers were less likely to receive a high level of education in their youth [62], which may have resulted in the abovementioned issue, since the workers with better educational background may be too young to be nominated as a potential crew leader at present in the Chinese context. Hence, further research on this issue is still needed given the improving education level of construction workers in China.

## 6. Conclusions

Taking scaffolders as an example, we proved the existence of opinion leaders in construction teams in the Chinese construction industry. Their influence was partly validated by different aspects of safety-related performance of their crews after a controlled behavioral intervention trial. Despite the proportions of opinion leaders within the surveyed construction crews varying on different scales of worksites, the influence of opinion leaders was mainly reflected in two kinds of workers: foremen and seasoned workers. However, we noticed that the seasoned worker-type leader needed to satisfy a certain tenure requirement on site, whereas foremen did not. This may be attributed to the different sources of their influence and the way they exert their influence. An unexpected finding in the study was that a worker’s practical skills and familiarity with the respondents seemingly played a more important role in the formulation of opinion leaders among construction workers in China. These findings not only contribute to the body of knowledge about social interaction among workers and resulting changes in behavior, but also provide guidance for identifying key people among workers for implementing more effective behavior-based safety practices.

Note that the method through which we selected an opinion leader was derived from the sociometric perspective and mainly concentrated on safety-related advice and knowledge among workers. As such, further research using other selection methods and different definitions of how to identify an opinion leader are still needed to consolidate our findings. In addition, with respect to the proposed method, the underlying reasons for the differences between ordinary information agents and opinion leaders, as well as the mechanism of how opinion leaders build and exert their influences on sites, especially for leaders without authorized power, are still unknown. In the future, with more objective and automated worker monitoring measures, our work can be extended to other workers from different work trades, regions, and cultural backgrounds, and more individual characteristics can be introduced to gain a more comprehensive understanding of this special role in construction crews.

## Figures and Tables

**Table 1:** Characteristics of the participants.

Variables	*N*	%
**Sex**		
Male	545	93
Female	41	7
**Educational level (Years)**		
>9	94	16
6–9	440	75
<6	52	9
**Status**		
Worker	557	95
Foreman	29	5
**Years of work experience**		
>20	404	69
10–20	100	17
5–10	58	10
<5	24	4
**Team tenure**		
>6 m	194	33
3–6 m	187	32
<3 m	205	35

**Table 2:** Checklist for worker unsafe behaviors observation.

Checklist for Worker Unsafe Behaviors Observation			
Observer:	Video Record Date:	Video Record Time:	
Instructions: For each observed activity, draw a small vertical line ‘‘l” in the column Safe behaviors if the activity is carried out in a safe way and in the column Unsafe behaviors if the activity is carried out in an unsafe way and may cause an incident. In the Notes column, it is possible to write additional information regarding the observed activities (e.g., type of behavior, the position of the scene in the video record)			
**Critical Issues**	**Safe**	**Unsafe**	**Notes**
Working in sleety weather without non-slipping shoes			
Working more than 2 m high without wearing a harness			
Climbing a scaffold without protection			
Standing in a location without sufficient scaffold floor			
Throwing member bars from a height during dismantling			
Stacking a lot of material on the scaffolds			

**Table 3:** Network metrics for the scale of the different project.

Sociometric Measures	All Respondents	Respondents of Network Scale		
		10–20	20–30	>30
Degree centrality—Question 1, mean	0.104	0.107	0.101	0.103
Degree centrality—Question 2, mean	0.116	0.113	0.108	0.121
Degree centrality—Question 3, mean	0.102	0.104	0.102	0.098
Degree centrality score, mean	0.104	0.102	0.104	0.106

**Table 4:** Project with identifiable opinion leader using 0.20 cut-points.

Network Scale	Project	Survey Participants	Foreman	Worker	Nomination Score Cutoff > 0.20	
					Foreman	Worker
>30	6	246	11	235	7 (63.6%)	13 (5.5%)
20–30	9	248	13	235	11 (84.6%)	21 (8.5%)
10–20	5	92	5	87	5 (100%)	12 (13.7%)

**Table 5:** Randomization checks of the variables measured by control and intervention condition.

Results	Sex		Education Level (Year)			Status		Work Experience (Year)				Tenure (Month)		
	M	F1	<6	6–9	>9	W	F2	<5	5–10	10–20	>20	<3	3–6	>6
Intervention	267	23	23	219	44	275	14	13	31	48	198	105	90	95
Control	278	18	29	221	50	282	15	11	27	52	206	100	97	99
χ2 value or t value	0.77		0.82			0.13		0.55				0.48		
*p* value	0.38		0.67			0.91		0.91				0.79		

Notes: M: number of male workers; F1: number of female workers; W: number of common workers; F2: number of foremen.

**Table 6:** Improvement of SI in six unsafe behaviors after the intervention.

Unsafe Behaviors	Intervention Groups			Control Groups			χ2 Value	*p* Value
	a (%)	b (%)	c (%)	a (%)	b (%)	c (%)		
Working in sleety weather without non-slipping shoes	54.6	83.5	28.9	55.3	62.5	7.2	212.07	0.00
Working more than 2 m high without harness	58.8	85.7	26.9	58.5	65.7	7.2	196.11	0.00
Climbing a scaffold without protection	55.4	73.5	18.1	56.2	63.5	7.3	61.73	0.00
Standing in a location without sufficient scaffold floor	77.4	84.2	6.8	76.2	82.2	6	1.38	0.24
Throwing member bars from height during dismantling	80.4	85.5	5.1	79.6	83.4	3.8	3.16	0.08
Stacking materials on scaffolds	62.5	75.2	12.7	60.8	65.2	4.4	56.72	0.00
Overall	62.5	83.4	20.9	61.8	67.3	5.5	142.36	0.00

Notes: a: Safety Index before intervention; b: Safety Index after intervention; c: Safety Index change.

**Table 7:** Boolean matrix of scaffolder’s association rule mining.

Participants	Sex		Education Level (Years)			Status	
	Male	Female	<6	6–9	>9	Leader	Other
**Worker 1**	1	0	0	0	1	1	0
**Worker 2**	1	0	1	0	0	0	1
**Worker 3**	1	0	0	1	0	0	1
**Worker 4**	0	1	0	1	0	0	1
**…**	…	…	…	…	…	…	…
**Worker *n***	0	1	0	1	0	0	1

**Table 8:** Categories of contributory factors: association rules. S: support (percentage of the entire data set covered by the rule); C: confidence (proportion of the number of examples that fit the right side among those that fit the left side).

Rule ID	Association Rule		S (%)	C (%)
	Antecedent	Consequent		
1	Foreman = 1	Leader	9.1	66.667
2	work experience 20 years = 1	Leader	7.2	88.8
	Team tenure			
	6 months = 1			

## References

[1] Pinto A., Nunes I.L., Ribeiro R.A. (2011). Occupational Risk Assessment in Construction Industry–Overview and Reflection. Saf. Sci..

[2] Shin M., Lee H.S., Park M., Moon M., Han S. (2014). A System Dynamics Approach for Modeling Construction Workers’ Safety Attitudes and Behaviors. Accid. Anal. Prev..

[3] Kachan D., Fleming L.E., LeBlanc W.G., Goodman E., Arheart K.L., Caban-Martinez A.J., Clarke T.C., Ocasio M.A., Christ S., Lee D.J. (2012). Worker Populations at Risk for Work-Related Injuries across the Life Course. Am. J. Ind. Med..

[4] Dong X.S., Wang X., Daw C., Ringen K. (2011). Chronic Diseases and Functional Limitations among Older Construction Workers in the United States: A 10-Year Follow-up Study. J. Occup. Environ. Med..

[5] Mohamed S., Ali T.H., Tam W.Y.V. (2009). National Culture and Safe Work Behaviour of Construction Workers in Pakistan. Saf. Sci..

[6] Lingard H., Rowlinson S. (1998). Behaviour-Based Safety Management in Hong Kong’s Construction Industry: The Results of a Field Study. Constr. Manag. Econ..

[7] Dingsdag D.P., Biggs H.C., Sheahan V.L. (2008). Understanding and Defining Oh&S Competency for Construction Site Positions: Worker Perceptions. Saf. Sci..

[8] Forteza F.J., Carretero-Gomez J.M., Sese A. (2017). Occupational Risks, Accidents on Sites and Economic Performance of Construction Firms. Saf. Sci..

[9] Ibarrondo-Dávila M.P., López-Alonso M., Rubio-Gámez M.C. (2015). Managerial Accounting for Safety Management. The Case of a Spanish Construction Company. Saf. Sci..

[10] Zhou Z., Goh Y.M., Li Q. (2015). Overview and Analysis of Safety Management Studies in the Construction Industry. Saf. Sci..

[11] Hinze J. (1981). Human Aspects of Construction Safety. J. Constr. Div..

[12] Sunindijo R.Y., Zou P.X.W. (2011). Political Skill for Developing Construction Safety Climate. J. Constr. Eng. Manag..

[13] Fang D.P., Xie F., Huang X.Y., Li H. (2004). Factor Analysis-Based Studies on Construction Workplace Safety Management in China. Int. J. Proj. Manag..

[14] Martin H., Lewis T.M. (2013). Pinpointing Safety Leadership Factors for Safe Construction Sites in Trinidad and Tobago. J. Constr. Eng. Manag..

[15] Rowlinson S., Mohamed S., Lam S.W. (2003). Hong Kong Construction Foremen’s Safety Responsibilities: A Case Study of Management Oversight. Eng. Constr. Archit. Manag..

[16] Li Q., Ji C., Yuan J., Han R. (2017). Developing Dimensions and Key Indicators for the Safety Climate within China’s Construction Teams: A Questionnaire Survey on Construction Sites in Nanjing. Saf. Sci..

[17] Chiaburu D.S., Harrison D.A. (2008). Do Peers Make the Place? Conceptual Synthesis and Meta-Analysis of Coworker Effects on Perceptions, Attitudes, Ocbs, and Performance. J. Appl. Psychol..

[18] Brondino M., Silva S.A., Pasini M. (2012). Multilevel Approach to Organizational and Group Safety Climate and Safety Performance: Co-Workers as the Missing Link. Saf. Sci..

[19] Schwatka N.V., Rosecrance J.C. (2016). Safety Climate and Safety Behaviors in the Construction Industry: The Importance of Co-Workers Commitment to Safety. Work.

[20] Chinowsky P.S., Diekmann J., O’Brien J. (2009). Project Organizations as Social Networks. J. Constr. Eng. Manag..

[21] Zohar D. (2010). Thirty Years of Safety Climate Research: Reflections and Future Directions. Accid. Anal. Prev..

[22] Liao P.C., Lei G., Fang D., Liu W. (2014). The Relationship between Communication and Construction Safety Climate in China. KSCE J. Civ. Eng..

[23] Chinowsky P., Diekmann J., Galotti V. (2008). Social Network Model of Construction. J. Constr. Eng. Manag..

[24] Buller D., Buller M.K., Larkey L., Sennott-Miller L., Taren D., Aickin M., Wentzel T.M., Morrill C. (2000). Implementing a 5-a-Day Peer Health Educator Program for Public Sector Labor and Trades Employees. Health Educ. Behav..

[25] Valente T.W., Pumpuang P. (2007). Identifying Opinion Leaders to Promote Behavior Change. Health Educ. Behav..

[26] Moreno J.L., Jennings H.H. (1938). Statistics of Social Configurations. Sociometry.

[27] Cartwright D., Harary F. (1956). Structural Balance: A Generalization of Heider’s Theory. Psychol. Rev..

[28] Freeman L.C. (1978). Centrality in Social Networks Conceptual Clarification. Soc. Netw..

[29] Valente T.W., Hoffman B.R., Ritt-Olson A., Lichtman K., Johnson C.A. (2003). Effects of a Social-Network Method for Group Assignment Strategies on Peer-Led Tobacco Prevention Programs in Schools. Am. J. Public Health.

[30] Keating N.L., Ayanian J.Z., Cleary P.D., Marsden P.V. (2007). Factors Affecting Influential Discussions among Physicians: A Social Network Analysis of a Primary Care Practice. J. Gen. Intern. Med..

[31] Sales A.E., Estabrooks C.A., Valente T.W. (2010). The Impact of Social Networks on Knowledge Transfer in Long-Term Care Facilities: Protocol for a Study. Implement. Sci..

[32] Koltsova O., Koltcov S., Nikolenko S. (2016). Communities of Co-Commenting in the Russian Livejournal and Their Topical Coherence. Internet Res..

[33] Starkey F., Audrey S., Holliday J., Moore L., Campbell R. (2009). Identifying Influential Young People to Undertake Effective Peer-Led Health Promotion: The Example of a Stop Smoking in Schools Trial (Assist). Health Educ. Res..

[34] Kaiser C., Kröckel J., Bodendorf F. (2013). Simulating the Spread of Opinions in Online Social Networks When Targeting Opinion Leaders. Inf. Syst. E-Bus. Manag..

[35] Allison L., Kaminsky J. (2017). Safety Communication Networks: Females in Small Work Crews. J. Constr. Eng. Manag..

[36] Alsamadani R., Hallowell M.R., Javernick-Will A., Cabello J. (2013). Relationships among Language Proficiency, Communication Patterns, and Safety Performance in Small Work Crews in the United States. J. Constr. Eng. Manag..

[37] Wehbe F., Al Hattab M., Hamzeh F. (2016). Exploring Associations between Resilience and Construction Safety Performance in Safety Networks. Saf. Sci..

[38] Alsamadani R., Hallowell M., Javernick-Will A.N. (2013). Measuring and Modelling Safety Communication in Small Work Crews in the Us Using Social Network Analysis. Constr. Manag. Econ..

[39] Ahn S., Lee S., Steel R.P. (2013). Effects of Workers’ Social Learning: Focusing on Absence Behavior. J. Constr. Eng. Manag..

[40] Gellatly I.R., Luchak A.A. (1998). Personal and Organizational Determinants of Perceived Absence Norms. Hum. Relat..

[41] Choi B., Ahn S., Lee S. (2017). Construction Workers’ Group Norms and Personal Standards Regarding Safety Behavior: Social Identity Theory Perspective. J. Manag. Eng..

[42] Andersen L.P., Karlsen I.L., Kines P., Joensson T., Nielsen K.J. (2015). Social Identity in the Construction Industry: Implications for Safety Perception and Behaviour. Constr. Manag. Econ..

[43] Albert A., Hallowell M.R. (2017). Modeling the Role of Social Networks on Hazard Recognition and Communication. Pract. Period. Struct. Des. Constr..

[44] Fang D., Wu C., Wu H. (2015). Impact of the Supervisor on Worker Safety Behavior in Construction Projects. J. Manag. Eng..

[45] Gambatese J., AlOmari K. (2016). Degrees of Connectivity: Systems Model for Upstream Risk Assessment and Mitigation. Accid. Anal. Prev..

[46] Li H., Lu M., Hsu S.C., Gray M., Huang T. (2015). Proactive Behavior-Based Safety Management for Construction Safety Improvement. Saf. Sci..

[47] Kravitz R.L., Krackhardt D., Melnikow J., Franz C.E., Gilbert W.M., Zach A., Paterniti D.A., Romano P.S. (2003). Networked for Change? Identifying Obstetric Opinion Leaders and Assessing Their Opinions on Caesarean Delivery. Soc. Sci. Med..

[48] DePasquale J.P., Geller E.S. (1999). Critical Success Factors for Behavior-Based Safety: A Study of Twenty Industry-Wide Applications. J. Saf. Res..

[49] Guo S.Y., Ding L.Y., Luo H.B., Jiang X.Y. (2016). A Big-Data-Based Platform of Workers’ Behavior: Observations from the Field. Accid. Anal. Prev..

[50] Cocca P., Marciano F., Alberti M. (2016). Video Surveillance Systems to Enhance Occupational Safety: A Case Study. Saf. Sci..

[51] Bastian M., Heymann S., Jacomy M. Gephi: An Open Source Software for Exploring and Manipulating Networks. Proceedings of the Third International ICWSM Conference.

[52] Komaki J.L. (1998). When Performance Improvement Is the Goal: A New Set of Criteria for Criteria. J. Appl. Behav. Anal..

[53] Saari J., Näsänen M. (1989). The Effect of Positive Feedback on Industrial Housekeeping and Accidents; a Long-Term Study at a Shipyard. Int. J. Ind. Ergon..

[54] Mitropoulos P., Memarian B. (2012). Team Processes and Safety of Workers: Cognitive, Affective, and Behavioral Processes of Construction Crews. J. Constr. Eng. Manag..

[55] Lingard H.C., Cooke T., Blismas N. (2009). Group-Level Safety Climate in the Australian Construction Industry: Within-Group Homogeneity and between-Group Differences in Road Construction and Maintenance. Constr. Manag. Econ..

[56] Hofmann D.A., Morgeson F.P. (1999). Safety-Related Behavior as a Social Exchange: The Role of Perceived Organizational Support and Leader–Member Exchange. J. Appl. Psychol..

[57] Uwakweh B.O. (2005). Effect of Foremen on Construction Apprentice. J. Constr. Eng. Manag..

[58] Kaskutas V., Buckner-Petty S., Dale A.M., Gaal J., Evanoff B.A. (2016). Foremen’s Intervention to Prevent Falls and Increase Safety Communication at Residential Construction Sites. Am. J. Ind. Med..

[59] Kaskutas V., Dale A.M., Lipscomb H., Evanoff B. (2013). Fall Prevention and Safety Communication Training for Foremen: Report of a Pilot Project Designed to Improve Residential Construction Safety. J. Saf. Res..

[60] Fang D., Chen Y., Wong L. (2006). Safety Climate in Construction Industry: A Case Study in Hong Kong. J. Constr. Eng. Manag..

[61] Zhang L., Liu Q., Wu X., Skibniewski M.J. (2016). Perceiving Interactions on Construction Safety Behaviors: Workers’ Perspective. J. Manag. Eng..

[62] Xia N., Wang X., Griffin M.A., Wu C., Liu B. (2017). Do We See How They Perceive Risk? An Integrated Analysis of Risk Perception and Its Effect on Workplace Safety Behavior. Accid. Anal. Prev..

